# Ventilator settings and outcome of respiratory failure in paraquat-induced pulmonary injury

**DOI:** 10.1038/s41598-019-52939-3

**Published:** 2019-11-12

**Authors:** Seyedehparvin Khazraei, Sayed Mahdi Marashi, Hossein Sanaei-Zadeh

**Affiliations:** 1 Student Research Committee, Shiraz University of Medical Sciences, Shiraz, Iran; 2 Blood Transfusion Research Center, High Institute for Research and Education in Transfusion Medicine, Tehran, Iran; 3 Emergency Room, Division of Medical Toxicology, Hazrat Ali-Asghar (p) Hospital, Shiraz University of Medical Sciences, Shiraz, Iran

**Keywords:** Prognosis, Signs and symptoms

## Abstract

Paraquat is a nonselective contact herbicide that has significant importance in clinical toxicology due to its high mortality rate. The cause of mortality in the acute phase of poisoning is a multi-organ failure while in the sub-acute phase is alveolar injury and lung fibrosis. The aim of this study was to evaluate the advantages and drawbacks of mechanical ventilation (MV) in paraquat-induced pulmonary injury and its consequential respiratory failure (PIPI-CRF). This retrospective descriptive analytical study was done to investigate the outcome of patients who had developed PIPI-CRF and underwent conventional treatments with invasive MV in three teaching hospitals in Shiraz, Iran, from March 2010 to February 2015. In total, 44 patients (mean age of 27.9 ± 9.98 years) had undergone MV due to PIPI-CRF. None of the patients had a successful wean off from the ventilator. Although all the patients’ were on aggressive life support and full efforts to resuscitate were carried out in case of cardiac arrest, all of them expired. We suggest that in the case of conventional treatment of paraquat poisoning, only noninvasive ventilation should be applied. However, considering the chance of patient’s survival performing novel treatments, such as extracorporeal membrane oxygenation (ECMO), lung protective ventilation with optimal positive end-expiratory pressure (PEEP) could be applied only in such circumstances.

## Introduction

Paraquat (1, dimethyl-4,4-bipyridylium dichloride-1) is the second bestselling herbicide, which is available in the form of 20% solution^1^. In humans, the most common way for poisoning is through ingestion with the aim to commit suicide; however, the other methods such as injection and respiration as well as cutaneous and mucosal contact have been also reported^2^. In the case of paraquat poisoning with the ingestion of more than 20 mg/kg, rapid multi-organ failure occurs and the patient expires within 2–3 days. In the case of poisoning with a lower amount, the patient might survive the acute phase by supportive care^3^. In the acute phase, the polyamine reuptake system, which is mainly expressed in the membrane of type II pneumocytes, attracts paraquat ions actively against plasma gradient. Consequently, paraquat concentration in the lung reaches 10 times more than the plasma compartment^4–8^. Hence, progressive lung fibrosis is possible even after relative recovery from acute phase^3,4^. Lung fibrosis usually occurs in patients who consumed more than 10 ccs of 20% solution. Some researchers believe that using immunosuppressive therapy like pulses of methylprednisolone and cyclophosphamide within the first few days can reduce the number of patients suffering from severe lung fibrosis^9,10^. Following lung fibrosis, a decrease in arterial O_2_ saturation and tissue hypoxia occurs. In the end stage, the patients are usually admitted to intensive care units (ICU), where they are placed under mechanical ventilation (MV)^2^.

According to previous studies, none of the therapeutic measurements was effective in preventing lung fibrosis or curing paraquat-induced pulmonary injury that had led to respiratory failure (PIPI-CRF). Most studies represent poor prognosis that ultimately leads to death within 2–3 weeks after poisoning, but MV as the mere supportive measure are still being used^2,3,11,12^.

The current study aimed to investigate the advantages and disadvantages of using MV for patients suffering from PIPI-CRF admitted to ICUs of three Shiraz teaching hospitals from March 2010 to February 2015.

## Methods

### Sample sources

In this retrospective descriptive analytical study, medical records of all paraquat-intoxicated patients who were under MV were reviewed at three teaching hospitals in Shiraz, Iran, from March 2010 to February 2015. Paraquat poisoning diagnosis was based on confessing to ingestion or from the label of toxin, observed by the emergency staff or medical doctors. Respiratory failure was defined by the deterioration of respiratory function, exacerbation of dyspnea and decrease in arterial O_2_ saturation to less than 80%, even after O_2_ therapy with a mask or continuous positive airway pressure (CPAP) therapy. For these patients, invasive MV through the endotracheal tube was performed.

Patients who had taken other medications or other types of poisons for suicide and those who had a past medical history of collagen vascular diseases, drug-induced pulmonary diseases, and radiation, cardiac, pulmonary, renal, or hepatic diseases were excluded from the study.

Data were obtained manually from the patient’s medical charts, which include age, gender, performance of hemodialysis or charcoal hemoperfusion within the first 6 hours following paraquat ingestion, duration of hospitalization prior to respiratory distress, incidence of CPAP and high flow O_2_ therapy before intubation, incidence of intubation, need for MV, mode of ventilator support, duration of MV, interval time between paraquat ingestion to respiratory failure, immunosuppressive or corticosteroid therapy for preventing pulmonary fibrosis, arterial blood gas analysis (within 6 h after MV), ventilator parameters after MV (i.e., tidal volume (TV), level of positive and expiratory pressure, plateau and peak airway pressures), body weight, demand for use of catecholamine medications following intubation, successful weaning off from MV (extubation or spontaneous respiration at least for 24 h with noninvasive positive pressure ventilation), performance of extracorporeal membrane oxygenation (ECMO), hospitalization time, survival time and cause of patient’s death. According to Saydain *et al*., multi-organ failure was defined as failure of two or more vital organs^8^.

### Statistical analysis

The data were analyzed using SPSS version 22 (SPSS Inc, Chicago, IL). Quantitative statistics are presented as mean ± standard deviation (SD) and qualitative statistics are reported as a percentage. A significant difference between groups was tested using a t-test for continues outcomes and *χ*^2^ test for categorical. The significance level was considered to be *P* < 0.05.

This study was approved by the regional scientific ethics committee of Shiraz University of Medical Sciences. This article is based on a research project guided by the second author (proposal #10521- 01- 01-94 approved on July 4, 2016). All methods were performed in accordance with the relevant guidelines and regulations. Furthermore, informed consent was obtained from all participants’ legal guardians.

## Results

In this study, a total of 45 patients with respiratory failure following paraquat poisoning within 5 years were evaluated. One case was excluded from the study due to simultaneous consumption of high dose methadone with paraquat. Finally, data of 44 patients were extracted from their medical records. Table 1 shows the patients demographic characteristics.

**Table 1:** Baseline demographics of the study subjects.

Demographic characteristics	Number (% of patients)
**Gender**	
Male	35(79.5)
Female	9(20.5)
Age (years) (mean ± SD)	27 ± 9.98
**Duration of hospitalization prior to respiratory distress**	
1–6 days	39(86.7)
7–13 days	4(8.9)
More than 14 days	1(2.2)
Mean(days)	2.78 ± 3.18
Time interval	3 (h)-15 (days)
**Duration of mechanical ventilation**	
Less than 2 days	32(71.1)
3–6 days	9(20)
More than 6 days	3(6.7)
Mean(days)	6.73 ± 5.73
Time interval	5 (h)-27 (days)

None of the patients had hemodialysis or charcoal hemoperfusion within the first 6 h following paraquat ingestion. The reason for not performing charcoal hemoperfusion was the lack of hospital facilities in Shiraz teaching hospitals; while the reason for postponing hemodialysis was waiting for checking viral markers report.

Combination therapy of methylprednisolone (at least 1500 mg within 3 days) and cyclophosphamide pulses (at least 1000 mg within 2 days) was administrated for 11 patients (25%) from the first day of admission. In 20 patients (45.5%), only corticosteroid therapy with methylprednisolone pulses was administrated. In 5 patients (11.4%), only dexamethasone 8 mg every 8 h was administrated.

Nine patients (20.5%) went under MV within the first 12 h, due to progressive respiratory failure. Mean interval time between paraquat ingestion to respiratory failure was 2.78 ± 3.13 days (3 h to 15 days). In 31 (70.5%) patients, before intubation, CPAP therapy and high flow O_2_ therapy were done. Eventually, due to progressive respiratory failure and decrease of O_2_ saturation, all of the patients underwent intubation. Following the intubation, all patients were sedated and underwent volume assist-control ventilation in the supine position. Mean TV was 6.97 ± 0.38 ml/kg and mean positive end-expiratory pressure was 5.38 ± 1.84 cm H_2_O, respectively. It has to be noted that only the data of 39 patients were available and in all cases weight were based on doctors’ estimation of the patients’ weight. In most cases, data changes in plateau pressure and peak airway pressure were not available. Blood gas analyses within 6 h after MV were the representation of significant hypoxia and mild hypocapnia (Mean PaO2/FiO2 ratio: 211.22 ± 77.33 and mean PaCO_2_: 33.0 ± 9.46 mm Hg; only the data of 26 patients was available). ECMO was not performed due to the lack of hospital facilities.

Mean of hospitalization time was 5.33 ± 6.05 days (10 h-29 days) while the mean of placing under MV was 2.55 ± 4.12 days (2 h-24 days). No patient was successfully weaned off from the ventilator. Aggressive life support and full effort resuscitation in the case of cardiac arrest was done for all the patients, however, all of them expired. Mean survival time for those who developed respiratory failure and were placed under MV within the first 48 h vs those who underwent MV after the second day was significantly lower (1.13 ± 1.27 vs 5.03 ± 5.96 days; p = 0.002). From 22 cases using catecholamine medications to sustain systolic blood pressure more than 90 mmHg, 20 expired within the first 24 h.

Common causes of death within the first 48 h after intubation were as follows: multi-organ failure; 15 cases, refractory hypoxia; 9 cases, pulmonary hemorrhage; 6 cases, disseminated intravascular coagulation; 1 case, and sepsis syndrome; 1 case. Total of 12 deaths after the third day was due to refractory hypoxemia.

## Discussion

The aim of this study was to prognosticate patients that developed PIPI-CRF and underwent MV. In this study, all patients expired due to respiratory failure without considering primary pulse therapy with corticosteroid or immunosuppressive drugs although being under respiratory and life support.

Lung fibrosis following parquet intoxication was the main cause of death during the sub-acute phase. To the best of our knowledge, this study is the first one that evaluates the outcome of MV in paraquat-intoxicated patients. One study claimed survival of paraquat-intoxicated patients who developed respiratory failure and required MV was 6.7% while for those without MV, it was 6.4%. However, some methodological biases should be taken into consideration. The result of this study was based on a nationwide population-based retrospective cohort study. Inclusion criteria were based on the diagnostic code “989.4”, which is the coding for “toxic effect of another pesticide that has not been classified, yet” and the authors considered performing hemoperfusion equal to paraquat intoxication^7^. Only one other review article reports the futility of respiratory support in patients with paraquat poisoning irrespective to any scientific background^13^.

Similarly, there is a belief that respiratory support is futile in patients with idiopathic pulmonary fibrosis, and most of the intensivists believe that only noninvasive ventilation must be applied^14^. The main difference between PIPI and other types of pulmonary fibrosis is the limitation in using O_2_ supplements in paraquat-intoxicated patients. Even though controlled hypoxia is not effective in preventing PIPI, it seems that oxygen supplementation can lead to progressive lung injuries^15^. Despite the mentioned differences between PIPI and IPF, most of the intensivists apply the same strategy to treat both.

Gaudry *et al*. used low TV ventilation (mean 5.9 ± 3 ml/kg) and reported the adjustment in the ventilator setting was associated with a better prognosis compared to previous studies that applied higher TV in IPF^16–19^. By adopting this strategy, some patients were weaned off successfully from the ventilator and discharged from ICU, which provided ample time for lung transplantation^16^. Mean of TV in the current study was (6.97 ± 0.38 ml/kg) while in our patients, this low TV did not lead to good prognosis. Despite the expected increase in the betterment of patients care, conventional treatments have not been able to hinder the progress of lung fibrosis and the mortality rate was still at 100%.

Within the last 20 years, several studies were conducted to investigate the outcome of lung protective ventilation methods^20–24^. For the first time, Amato *et al*. claimed that low TV and high PEEP levels could reduce mortality in patients with acute respiratory distress syndrome (ARDS)^21^. Then, the results of a large multicenter ARDS net trial showed that decreasing TV to 6 ml/kg instead of 12 ml/kg could reduce mortality up to 25% in the ARDS patients^25^. The reasoning for TV reduction goes back to the descriptive concept of the baby lung in ARDS, which explains physiologically, we face small lung rather than a stiff one, as assumed before^26^. Hence, this strategy needs more investigation toward using it in pulmonary fibrosis.

Fernandez-Perez *et al*. showed that in patients with chronic interstitial lung disease, the rise of PEEP leads to an inappropriate rise in peak and plateau airway pressures. This increase consequently results in a decrease in respiratory system compliance such that the patients exposed to PEEP of >10 cm H_2_O are at higher risk of mortality^27^. Similarly, in paraquat poisoning, disruption in gas transmission and increased lung stiffness occur with mechanisms like interstitial inflammation, peribronchial fibrosis, and interstitial fibrosis^28^. Therefore, as ARDS, paraquat intoxicated patients encounter the decreased aerated lung. In contrast to ARDS, lung edema does not have any role in the pathogenesis of PIPI. Accordingly, it seems that high PEEP strategy not only cannot lead to the betterment of oxygenation but also leads to lung overdistention and ventilation-induced lung injury. The mentioned consequences adequately explain the pathophysiology behind interstitial lung disease^27^. In our study, reduced PEEP to 5.38 ± 1.84 cm/H_2_O had no positive effect on prognosis. However, it is not clear whether we can confidently equate the effects of higher PEEP on patients with paraquat-induced lung injury to those with an acute exacerbation of interstitial lung disease. The effect of PEEP on lung recruitment and secondarily plateau and driving pressure depends on recruitable alveoli, which may be inherently different in an acute process versus a chronic, fibrotic process despite similar cellular pathophysiology. Moreover, the patients in this study did not have readily available peak, plateau, or driving pressures on which test this hypothesis.

As mentioned above, even new concepts of ventilation could not be effective in the prognosis of PIPI-CRF. Accordingly, our study confirms the study of Gawarammana and Buckley who reported that ventilatory support is futile in these cases^13^. Moreover, our study showed that conventional treatment and invasive ventilatory support are associated with death prognosis. Although there is no evidence to support that MV and conventional treatments are associated with a change in prognosis compared to non-invasive ventilation, managing patients with MV may be a lifesaving option if ECMO is available either as a bridge to recovery or lung transplantation. In this case, we suggest the application of lung protective ventilation with optimal PEEP. However, it should be noted that still there is no clear criteria to help decide on starting ECMO in PIPI. In contrast, initiation of ECMO for respiratory failure requires that the patient does not improve despite optimal, conventional mechanical ventilatory strategies. Additionally, the patient must have severe ARDS (PaO_2_/FiO_2_ is ≤100 mmHg on ventilator settings that include PEEP ≥ 5 cm H_2_O). However, in our study, the patients did not meet criteria for severe ARDS (mean PaO_2_/FiO_2_ ratio of 211.22 ± 77.33 and PEEP 5.38 ± 1.84) averagely.

It is possible that some new treatments like ECMO and administrating pirfenidone can open new horizons for treating paraquat poisoning. Recent studies have shown that performing ECMO to treat lung fibrosis could provide enough time for lung transplantation^16^. However, it is only Tang *et al*. who reported the effectiveness of ECMO in paraquat poisoning^29^. Considering this fact that lung transplantation is the mere curative treatment for the end stages PIPI-CRF^29,30^, ECMO can act as a bridge to lung transplantation after cleansing the body from paraquat.

Fuehner *et al*. showed that ECMO could also be applied in non-intubated patients^31^. Accordingly, through this method, adequate oxygenation can be provided without ventilator-induced lung injuries. Additionally, a window of opportunity is created for physicians to try other approaches to reduce the need for lung transplantation as a consequence of PIPI.

Pirfenidone is one of these new therapies. For the first time, in 2012, Seifirad *et al*. showed that pirfenidone decreases pulmonary fibrosis in a rat model of paraquat poisoning^32^. In the same year, Sanaei-Zadeh suggested the use of pirfenidone for preventing PIPI^11^. In a study published in 2017, simultaneous administration of pirfenidone and prednisolone reduced pulmonary fibrosis in a rat model^28^. Considering one experience, using pirfenidone alongside with other immunosuppressive agents can be effective in preventing PIPI. Sanaei-Zadeh and Marashi reported that adding pirfenidone to conventional treatments could prevent paraquat-induced pulmonary fibrosis (unpublished data). Although there is no controlled human study in this regard, we assume if a paraquat-intoxicated patient survives from acute phase, applying ECMO and treatment with pirfenidone alongside with other immunosuppressive agents might be effective in preventing the formation of a fibrotic lesions. Although these pieces of evidence are too feeble to support a recommendation, we assume it can establish a favorable basis for a future trial. Thus, we suggest lung protective ventilation with optimal PEEP, only in the cases who receive innovational therapies. Moreover, it must be considered that even in these cases, no safe setting has been introduced yet^16,33^.

Our study had some limitations. First of all, data were collected retrospectively and, in all of the cases, ventilator setup was adjusted based on the patient’s estimated weight.

Secondly, lack of accurate registration of plateau pressure, peak airway pressure, and change of setup according to clinical conditions led to the failure of evaluating alveolar distension, and its consequent lung injuries.

Thirdly, reasons behind the use of higher TV or PEEP were not registered in the medical files. Besides, ventilator setup, and the manner of using neuromuscular blockers (which plays an important role in reducing ventilator inducing lung injury) were not clear from medical reports.

## Conclusions

Results of this study showed that ventilatory support in the cases of respiratory failure following paraquat poisoning does not help their survival. Moreover, conventional therapies for preventing pulmonary fibrosis when patients develop respiratory distress cannot stop the injury trend. Performing conventional treatment and ventilatory support is associated with death prognosis; therefore, it seems that only noninvasive ventilation must be applied. However, some evidence shows that novel treatments like ECMO and prescribing pirfenidone to prevent PIPI-CRF can raise the chance of survival. Thus, prospective controlled trial studies to determine the efficacy of lung protective ventilation with optimal PEEP in patients under novel treatments must be conducted.
